# Investigating the Properties and Characterization of a Hybrid 3D Printed Antimicrobial Composite Material Using FFF Process: Innovative and Swift

**DOI:** 10.3390/ijms24108895

**Published:** 2023-05-17

**Authors:** Waleed Ahmed, Ali H. Al-Marzouqi, Muhammad Hamza Nazir, Tahir A. Rizvi, Essam Zaneldin, Mushtaq Khan, Muthanna Aziz

**Affiliations:** 1 Engineering Requirements Unit, College of Engineering, United Arab Emirates University, Al Ain P.O. Box 15551, United Arab Emirates; 2 Department of Chemical and Petroleum Engineering, College of Engineering, United Arab Emirates University, Al Ain P.O. Box 15551, United Arab Emirates; 3 Department of Microbiology & Immunology, College of Medicine & Health Sciences, United Arab Emirates University, Al Ain P.O. Box 15551, United Arab Emirates; 4 Zayed Center for Health Sciences, United Arab Emirates University, Al Ain P.O. Box 15551, United Arab Emirates; 5 Department of Civil and Environmental Engineering, College of Engineering, United Arab Emirates University, Al Ain P.O. Box 15551, United Arab Emirates; 6 Department of Mechanical Engineering, College of Engineering, United Arab Emirates University, Al Ain P.O. Box 15551, United Arab Emirates

**Keywords:** polylactide, hybrid composites, 3D printing, long-term antimicrobial efficiency, biodegradable

## Abstract

Novel strategies and materials have gained the attention of researchers due to the current pandemic, the global market high competition, and the resistance of pathogens against conventional materials. There is a dire need to develop cost-effective, environmentally friendly, and biodegradable materials to fight against bacteria using novel approaches and composites. Fused filament fabrication (FFF), also known as fused deposition modeling (FDM), is the most effective and novel fabrication method to develop these composites due to its various advantages. Compared to metallic particles alone, composites of different metallic particles have shown excellent antimicrobial properties against common Gram-positive and Gram-negative bacteria. This study investigates the antimicrobial properties of two sets of hybrid composite materials, i.e., Cu-PLA-SS and Cu-PLA-Al, are made using copper-enriched polylactide composite, one-time printed side by-side with stainless steel/PLA composite, and second-time with aluminum/PLA composite respectively. These materials have 90 wt.% of copper, 85 wt.% of SS 17-4, 65 wt.% of Al with a density of 4.7 g/cc, 3.0 g/cc, and 1.54 g/cc, respectively, and were fabricated side by side using the fused filament fabrication (FFF) printing technique. The prepared materials were tested against Gram-positive and Gram-negative bacteria such as *Escherichia coli* (*E. coli*), *Staphylococcus aureus* (*S. aureus*), *Pseudomonas aeruginosa* (*P. aeruginosa*), *Salmonella* Poona (*S.* Poona), and *Enterococci* during different time intervals (5 min, 10 min, 20 min, 1 h, 8 h, and 24 h). The results revealed that both samples showed excellent antimicrobial efficiency, and 99% reduction was observed after 10 min. Hence, three-dimensional (3D) printed polymeric composites enriched with metallic particles can be utilized for biomedical, food packaging, and tissue engineering applications. These composite materials can also provide sustainable solutions in public places and hospitals where the chances of touching surfaces are higher.

## 1. Introduction

The industrial production name for 3D printing is an additive manufacturing (AM) or additive layer manufacturing (ALM). A computer-controlled technique develops three-dimensional things by depositing materials, typically in layers. AM enables the fabrication of lighter, more substantial parts and systems that offer digital flexibility and efficiency to manufacturing operations through the precise deposit of layer upon layer of material. The term “additive manufacturing” (AM) involves several fabrication techniques. The most popular technique is material extrusion (ME), which consists of feeding a material filament into the extrusion system and heating it close to the polymer’s melting point. The end effector produces and fuses the new material layer to the older layer [1]. The simplicity of operation, safety, and waste reduction are considerably enhanced by producing metal components with ME. The drawbacks can be lessened by using ME technology to create reasonably priced metal components within a special research area that is carefully examined. This technique creates a metal–polymer composite (MPC) filament by melting a polymer matrix and metal powder together. Nano and ultrafiltration have been used to manufacture antimicrobial materials utilizing various processes (such as spray coating, dip coating, and spin coating). In order to produce complexly shaped materials with improved properties that are challenging to achieve through conventional fabrication techniques, 3D printing technology is currently attracting more interest in the development of antimicrobial materials for medical applications [2,3]. The CAD model is used to create 3D materials for this additive manufacturing process, and materials are deposed one layer at a time. The most popular technique for creating medical devices is fused filament manufacturing. This is the most effective technique since it can fabricate tiny mechanical parts and offers precise accuracy, which are useful for any modifications in the desired result, even during the production process. Different methods, including stereolithography, digital light processing, selective laser sintering, and fused filament fabrication, are currently used for 3D printing on a global scale [4,5]. According to the investigation by Vidakis et al. [6] on the antibacterial effectiveness of PLA/AgNp created using fused filament fabrication against two different types of common bacteria across various periods, the generated material significantly decreased the percentage of germs. Yang et al. [7] claimed a 90.43% reduction in the development of E. coli after testing the antibacterial effectiveness of wood plastic composites supplemented with copper-zinc alloy particles. According to the literature, 3D printed materials also have improved mechanical and thermal qualities as well as antibacterial effectiveness.

Antimicrobial materials are substances or surfaces that can inhibit the growth of various micro-organisms. These materials are often used in medical settings and consumer products to prevent the spread of infections and maintain cleanliness. Antimicrobial materials can be treated with antimicrobial agents or naturally occurring substances with antimicrobial properties. Examples of antimicrobial materials include copper, silver, and some polymers with built-in antimicrobial agents. The use of biodegradable alternatives made from renewable sources is becoming more popular due to the environmental concerns of non-renewable and non-biodegradable materials. It has become increasingly common to develop novel materials by blending two or more polymers in order to give additional characteristics that could not be obtained by utilizing a single polymer [8]. Compared to metals and glass, thermoplastic polymers have low process temperatures, adjustable barrier qualities, printability, heat seal ability, and simplicity of processing into various forms, all of which make them good candidates for designing different materials that can be used for multiple applications [9]. Polylactic acid (PLA), a polyester made from lactic acid (LA), which is derived from the fermentation of corn starch and other polysaccharides, is one of the most promising biodegradable thermoplastic materials [10]. Furthermore, it has been demonstrated that prepared and characterized PLA-based composites, when mixed with four different antimicrobial fillers (silver, hexadecylpyridinium, hexadecyltrimethylammonium bromides anchored on vermiculite, graphene oxide matrices in an amount of 1% wt), has proven significant antimicrobial properties concerning their antimicrobial character for possible use in the production of medical stents [11]. PLA can be easily modified by using different fillers to develop nano-composites with new physical, mechanical, and antimicrobial characteristics [12]. Several materials with proven antibacterial properties can be used as fillers, including chitosan, metal nanoparticles, their oxides, or ionic forms, such as silver, copper, iron oxides, zinc oxide, titanium oxide, calcium oxide, as well as carbon nanomaterials [13,14]. Numerous studies have investigated the antimicrobial properties of PLA through surface modification or composite synthesis utilizing an antimicrobial agent or a combination of different antimicrobial agents. Composites have been developed using various polymers and varying amounts of fillers tested for their antimicrobial anti-activities [15,16]. Among numerous metals, copper exhibits excellent antimicrobial properties and has been used for various medical applications since human civilization. In the late 19th and early 20th centuries, inorganic copper formulations have been widely used to treat diseases such as chronic adenitis, eczema, impetigo, scrofulosis, tuberculous, lupus, syphilis, anemia, chorea, and facial neuralgia [17]. Copper was still used as an antibacterial agent in 1932, even after antibiotics became widely accessible [18]. In healthcare settings, food processing facilities, and animal breeding facilities, antibiotic-resistant bacteria are pervasive due to the selective pressure that causes their spread. This has increased the need for various strategies to ward off harmful microbes. The Environmental Protection Agency (EPA) recognized over 300 different copper surfaces as antimicrobials in 2008, which boosted the study of metallic copper surfaces’ antibacterial qualities [19]. In general, copper and its particles have an efficient antimicrobial effect [20]. The efficiency of copper and its particles dramatically depends on their size compared to their concentration: the smaller the particle size, the greater the efficiency [21]. Brass and bronze are two commonly used copper alloys; the former is made of copper and zinc, while the latter is made of copper and other metals, usually tin, aluminum, nickel, and metalloids such as silicon [22]. Studies have revealed a clear relationship between copper concentration and antimicrobial efficacy and that copper alloys exhibit antimicrobial activity. The composition of metals can be changed to improve antibacterial activity and other properties. For example, brass’s copper-to-zinc ratio can be adjusted to make the surface hard for applications such as hospital furniture and doorknobs [23,24].

Due to its clean look, higher mechanical strength, effective biocompatibility, and corrosion-resistant nature, stainless steel is the metal that is most frequently used in healthcare settings. However, employing this metal has no built-in antibacterial benefits [25]. With its ability to self-sanitize, copper surfaces have the potential to reduce the spread of infections significantly [26]. Thus, as recently demonstrated in fruitful hospital studies, antimicrobial metallic copper surfaces are anticipated to offer protection against infectious bacteria by lowering surface contamination [27]. Furthermore, different strategies have been adopted to improve the surface characteristics of stainless steel by modifying its surface structure and other surface properties using various technologies [28]. Similarly, like stainless steel, aluminum does not possess any effective antibacterial properties, and bacteria can quickly grow on its surface [29].

Hybrid composites of these metallic particles were prepared using the printing technique, which is an efficient, cost-effective, and rapid method to introduce metal particles in the polymer matrix and can be enhanced by using natural material [30,31], where the recycled plastic waste can be used as a matrix to develop the antimicrobial composite [32]. Two or more non-conductive metallic materials could be used in different combinations, such as 3D printed strips produced side by side or made by any other technology, particles mixed with polymer-based material. Moreover, one or more spray-based bimetallic compounds can be applied on conductive insulated substrates. The developed bimetallic compound can be sprayed based on two or more materials and can be used on conductive or insulated substrates. Furthermore, bimetallic materials can be used for any sort of base material with various types and properties, including, but not limited to, flexible, textiles, such as face masks, fabrics, clothes, shoes, etc. The concept can be implemented by employing two or more known metallic materials while considering the efficacy may vary based on the chosen metallic materials related to each material’s electromotive force. Because of their adaptability and being simple to use, they can be applied to various geometric shapes to fit a wide range of applications, including, but not limited to, applications needing rapid and continuous sanitization in medical and public places, such as window handles and door locks, knobs/handles, water tap, and so on. It can be used with 3D printing technology to produce bimetallic compounds by mixing or spraying technology for antimicrobial surfaces while printing. Such a strategy allows for achieving antimicrobial properties for identified surfaces of complex shapes and geometries. The bimetallic compound can be sprayed or mixed during any fabrication process (e.g., fabricating construction material), or used to spray existing products exposed to frequent contamination (e.g., medical appliances). It is sustainable since it can be used to make antimicrobial face masks, fabrics, and clothes that can be used for an extended period without disposal, and it is washable without affecting the antimicrobial characteristics. Furthermore, it can be used to produce fabrics with antimicrobial properties by using any weaving type or shape, or even using any combination of different metallic non-conductive composite strings that can have antimicrobial properties. Moreover, it has self-sanitization properties that do not need further sanitization by any sanitizer, such as alcohol or ultraviolet (UV) light.

The present study aimed to produce the composites of PLA-Cu-SS and PLA-Cu-Al through 3D printing using fused filament fabrication. We demonstrate that the biocompatibility of PLA filaments was not altered after the extrusion process. This supports the idea that the positive post-processing modifications needed for customization can be easily accomplished. Furthermore, the effect of the antibacterial properties of these developed materials have been tested according to international standards and for different time intervals against various bacteria, such as *E. coli*, *S. aureus*, *P. aeruginosa*, *S. Poona*, and *Enterococci*, to establish the proof of principle and the extraordinary property of the rapid antimicrobial resistance. The results presented in this study showed that PLA-based 3D printed composites could be employed as potential materials for biomedical applications containing excellent antimicrobial activities. Overall, our results support the idea that such a technology could provide a perfect methodology to fabricate the surface of stainless steel and aluminum to enhance their antibacterial properties.

## 2. Results and Discussions

**Figure 1:**
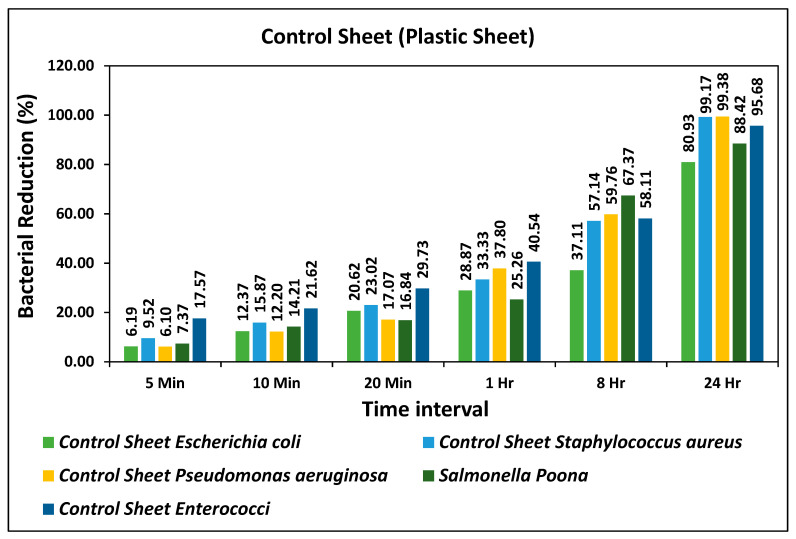
Bacterial reduction (%) on “Control Sheet” for five types of bacteria during different time intervals.

Two types of 3D printed polymeric composite sheets were developed by mixing a known composition, PLA-Cu-SS and PLA-Cu-Al, and labeled as samples 1 and 2, respectively. A commercial nylon sheet has been used as a reference sample to compare the antimicrobial results with prepared PLA-Cu-SS and PLA-Cu-Al composites [4]. The bacterial reduction (%) during different time intervals, i.e., 5 min, 10 min, 20 min, 1 h, 8 h, and 24 h, is given in Figure 1 for five types of Gram-positive and Gram-negative bacteria: *Escherichia coli*, *Staphylococcus aureus*, *Pseudomonas aeruginosa*, *Salmonella* Poona, and *Enterococci*. It can be seen from the graph that the bacterial reduction (%) is not significantly increased until 8 h, and still, more than 60% of *Escherichia coli* was present on the plastic surfaces; however, after 24 h the maximum reduction tendency was observed for all types of bacteria, i.e., more than 80%. The bacterial count of all bacteria on the control sheet during different time intervals is given in Table 1. The bacterial count was observed at a minimum after 24 h for *Staphylococcus aureus*, *Pseudomonas aeruginosa* (52 and 51, respectively); however, *Escherichia coli*, *Salmonella* Poona, and *Enterococci* bacteria were present in a large quantity on the control surface even after 24 h, as given in Table 1. The column “inoculum” in Table 1 shows the initial concentrations of all types of bacteria. The standards ISO 22196:2011 [33] and CCFRA:1:1:4:2003 [34] were followed for testing procedures and microbiology analytical methods [35,36].

**Figure 2:**
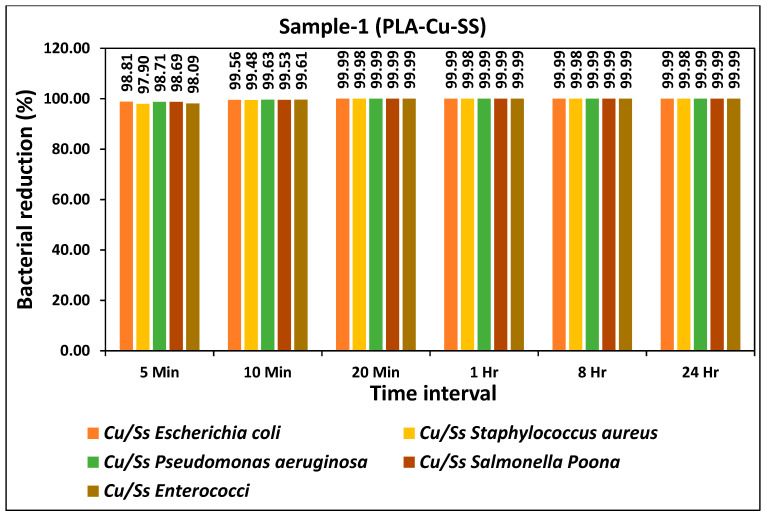
Bacterial reduction (%) on “PLA-Cu-SS Sheet” for five types of bacteria during different time intervals.

The bacterial reduction (%) of all types of bacteria during different time intervals for the prepared PLA-Cu-SS sheet is given in Figure 1. The prepared sheet showed excellent antibacterial efficiency and more than 95% reduction in bacterial activity for all types of bacteria was achieved just after 5 min as shown in Figure 2. Antibacterial efficiency was further tested for 10 min, 20 min, 1 h, 8 h, and 24 h and the results showed consistency as more than 99% antibacterial efficiency was achieved for all bacteria after 10 min. The bacterial count of all bacteria tested on PLA-Cu-SS sheet during different time intervals is given in Table 2. The bacterial count was reduced significantly from the initial concentration over time (5 min to 20 min); however, after 20 min, the maximum reduction in the bacterial count was achieved, and no further change was observed from 1 h to 24 h. The decrease in bacterial amount attributed to the higher amount of copper particles used in the present composites [37].

The results of the present study revealed excellent antimicrobial efficiency of PLA-Cu-SS composite sheets against five types of micro-organisms tested. Compared to the previously described study, the antimicrobial efficiency of PLA-Cu-SS sheets is better against Escherichia coli and Staphylococcus aureus, which reported only 60% antimicrobial efficiency after 24 h using a cellulose-based composite [38]. It has been shown that antimicrobial bimetallic polymer-based composites contain the superior characteristic of diminishing the bacteria on surfaces within a short time [39]. The excellent antibacterial properties originate from using a bimetallic non-conductive composite made from polymer-based materials. The voltage differential between at least two different non-conductive metallic composites (electromotive force) is proportional to the efficiency of the antibacterial characteristics [40].

**Figure 3:**
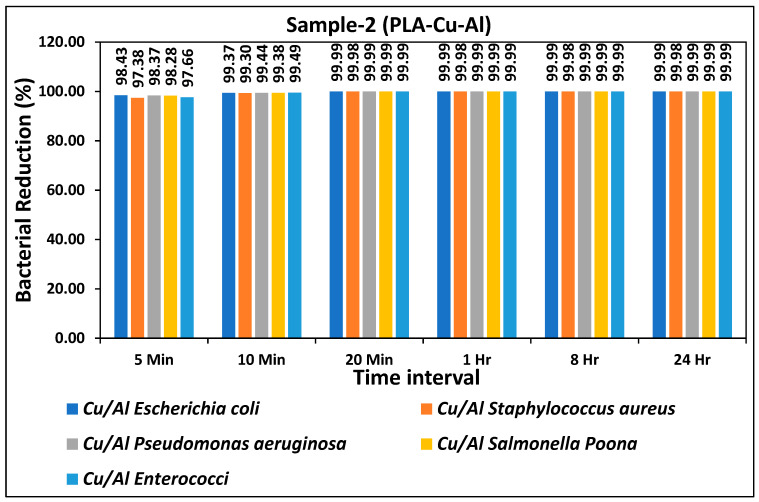
Bacterial reduction (%) on “PLA-Cu-Al” for five types of bacteria during different time intervals.

The bacterial reduction (%) of all types of bacteria during different time intervals for the prepared PLA-Cu-Al sheet is given in Figure 2. The prepared sheet showed excellent antibacterial efficiency and a more than 95% reduction in bacterial activity for all types of bacteria was achieved just after 5 min, as shown in Figure 3. Antibacterial efficiency was further tested for 10 min, 20 min, 1 h, 8 h, and 24 h, and results showed consistency, as more than 99% antibacterial efficiency was achieved for all bacteria just after 10 min. There was no significant difference between the two types of prepared sheets as both showed similar antibacterial efficiency for all bacteria, as shown in Figure 1 and Figure 2, respectively. The bacterial count of all bacteria on PLA-Cu-Al sheet during different time intervals is given in Table 3. The bacterial count was reduced significantly from the initial concentration over time (5 min to 20 min); however, after 20 min, the maximum reduction in the bacterial count was achieved, and no further change could be observed from 1 h to 24 h. There was a minor difference in the bacterial count for both sheets for 5–10 min, but after 20 min, the bacterial count for both sheets was the same as in Table 2 and Table 3, respectively. It can be seen from Table 2 and Table 3 that the PLA-Cu-SS sheet showed a better efficiency when compared to PLA-Cu-Al sheet but this change in efficiency was not significant.

In previous studies, N-methylene phosphonic acid chitosan, graphene sheets, and urea-derived graphitic carbon nitride sheets embedded with silver nanoparticles showed excellent antimicrobial agents against *Escherichia coli* and *Staphylococcus aureus* [41,42]. In most of the previous studies, the efficiency of prepared samples was tested against two types of bacteria, i.e., *Escherichia coli* and *Staphylococcus aureus*. In the present study, the prepared samples were tested against five types of bacteria during different time intervals. The results depicted that the antimicrobial efficiency of the prepared samples was much better in terms of time compared to previously reported studies where the developed material was tested against two microbes, i.e., *Escherichia coli* and *Staphylococcus aureus* [38,39].

**Figure 4:**
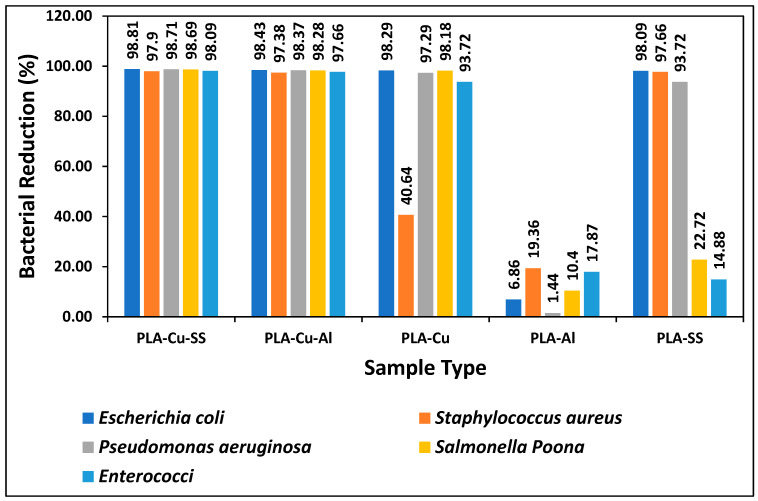
Comparison of antimicrobial efficiency of 3D printed PLA-Cu-SS and PLA-Cu-Al sheets with PLA-Cu, PLA-Al and PLA-SS against *Escherichia coli*, *Staphylococcus aureus*, *Pseudomonas aeruginosa*, *Salmonella* Poona and *Enterococci* for 5 min.

The comparison of the present study with the previously published data is given in Figure 3. Previously, three types of 3D printed composite sheets, i.e., PLA-Cu, PLA-Al, and PLA-SS were developed, and their antimicrobial efficiency was tested against five types of micro-organisms, i.e., “*Escherichia coli*, *Staphylococcus aureus*, *Pseudomonas aeruginosa*, *Salmonella* Poona and *Enterococci*” during different time intervals [4]. PLA-Cu exhibited 99.99% efficiency against all types of bacteria after 20 min. The other two samples (PLA-Al and PLA-SS) showed maximum efficiency after 8 h; however, the samples (PLA-Cu-SS and PLA-Cu-Al) presented in this study achieved the maximum antimicrobial efficiency of 98.43% and 99.99%, respectively, after 5 and 10 min, which is a much shorter time interval compared to the previously published study [4]. A comparison of antimicrobial efficiency of 3D printed PLA-Cu-SS and PLA-Cu-Al sheets with PLA-Cu, PLA-Al, and PLA-SS against *Escherichia coli*, *Staphylococcus aureus*, *Pseudomonas aeruginosa*, *Salmonella* Poona and *Enterococci* for 5 min is given in Figure 4.

**Figure 5:**
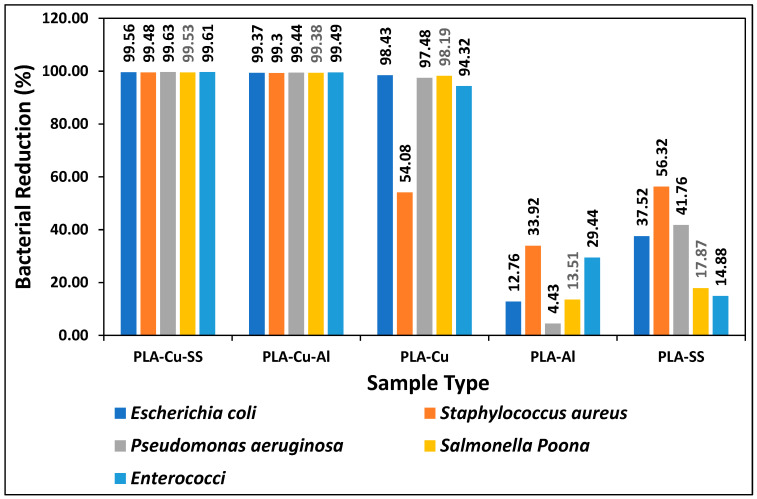
Comparison of antimicrobial efficiency of 3D printed PLA-Cu-SS and PLA-Cu-Al sheets with PLA-Cu, PLA-Al, and PLA-SS against *Escherichia coli*, *Staphylococcus aureus*, *Pseudomonas aeruginosa*, *Salmonella* Poona and *Enterococci* for 10 min.

It can be seen from Figure 3 that PLA-Al has the least antimicrobial efficiency against all types of bacteria after 5 min. It can be because aluminum and its alloys do not possess meaningful antimicrobial activity [29]. However, PLA-Cu sheet showed excellent antimicrobial efficiency of more than 90%, except for *Staphylococcus aureus*, which had an efficiency of 40.64%. Similarly, PLA-SS achieved more than 90% bacterial reduction except for *Salmonella* Poona and *Enterococci*. Furthermore, the 3D printed composites of PLA-Cu-Al and PLA-Cu-SS achieved more than 97% reduction after 5 min for all types of bacteria. The comparison of the samples prepared in the present study with the previous ones during a time interval of 10 min is given in Figure 5 [4].

Based on the above results, it can be concluded that the combined antimicrobial effect of 3D printed PLA-Cu-SS and PLA-Cu-Al sheets is much better than PLA-Cu, PLA-Al, and PLA-SS, as presented in the previous study [4].

### Agar Plate Diffusion Test

**Figure 6:**
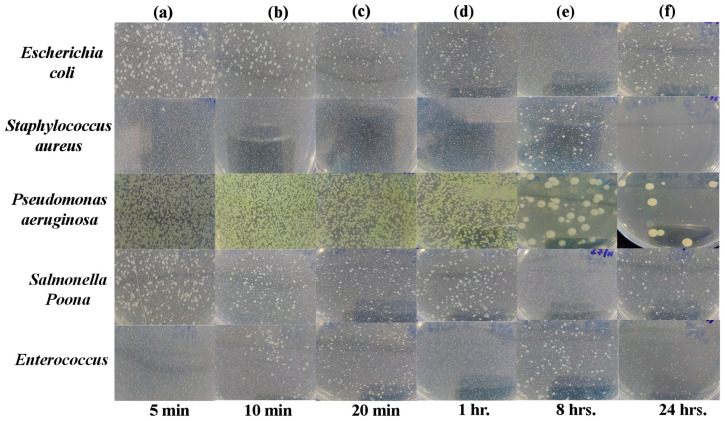
Images of Petri dishes used in agar diffusion method for “Control Sheet” against “*Escherichia coli*, *Staphylococcus aureus*, *Pseudomonas aeruginosa*, *Salmonella* Poona, and *Enterococci*” during different time intervals. (**a**) 5 min, (**b**) 10 min, (**c**) 20 min, (**d**) 1 h, (**e**) 8 h, and (**f**) 24 h.

Agar plate test allows fast identification with susceptibility, giving effective results and measurements of growth inhibition inflicted on a tested micro-organism [43]. Images of Petri dishes used in the agar diffusion method for the “Control Sheet” against “*Escherichia coli*, *Staphylococcus aureus*, *Pseudomonas aeruginosa*, *Salmonella* Poona and *Enterococci*” during different time intervals (i.e., 5 min, 10 min, 20 min, 1 h, 8 h, and 24 h) are given in Figure 6a–f. The results showed that the plastic control sheet does not have significant antibacterial efficiency against all tested bacteria, as they are still present in large amounts even after 24 h.

**Figure 7:**
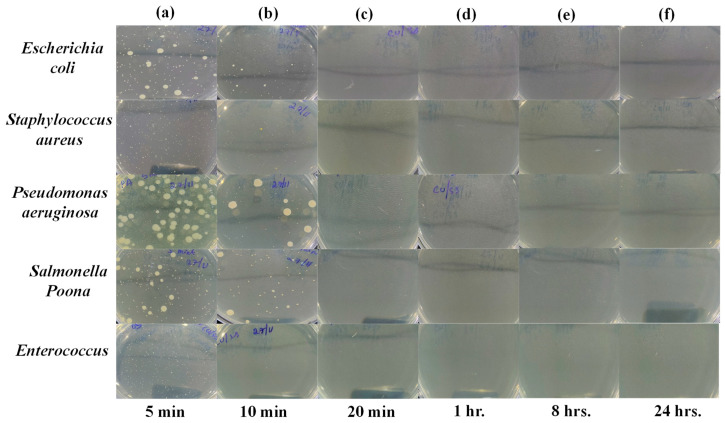
Images of Petri dishes used in agar diffusion method for “PLA-Cu-SS Sheet” against “*Escherichia coli*, *Staphylococcus aureus*, *Pseudomonas aeruginosa*, *Salmonella* Poona, and *Enterococci*” during different time intervals. (**a**) 5 min, (**b**) 10 min, (**c**) 20 min, (**d**) 1 h, (**e**) 8 h, and (**f**) 24 h.

However, as shown in Figure 6 and Figure 7, after exposure to the PLA-Cu-SS sheet and PLA-Cu-Al sheet, the presence of bacteria started declining, demonstrating their effective antibacterial activity. The antibacterial activity of these composites can be due to copper and aluminum nanoparticles’ which can coagulate proteins and hinder the growth of bacteria [44]. Furthermore, bacterial membrane proteins attached to the copper and aluminum nanoparticles may interfere with the production of peptidoglycan and impede the construction of cell walls. It has been suggested that this mechanism can efficiently reduce bacterial growth (e.g., *Escherichia coli*, *Staphylococcus aureus*, *Pseudomonas aeruginosa*, *Salmonella* Poona, and *Enterococci*) [45]. After 20 min, the PLA-Cu-SS sheet exhibited maximum antibacterial efficiency (99.99%) against all tested bacteria. Similar results were obtained for the PLA-Cu-Al sheet after 20 min against tested bacteria. Images of Petri dishes used in the agar diffusion method for the “PLA-Cu-SS Sheet” against “*Escherichia coli*, *Staphylococcus aureus*, *Pseudomonas aeruginosa*, *Salmonella* Poona, and *Enterococci*” during different time intervals (i.e., 5 min, 10 min, 20 min, 1 h, 8 h, and 24 h) are given in Figure 7a–f. It can be observed from Figure 7 that no bacteria were left after 20 min on the Petri dish, and no further growth could be observed even after 24 h, depicting that the PLA-Cu-SS sheet has excellent efficiency against all types of tested bacteria.

**Figure 8:**
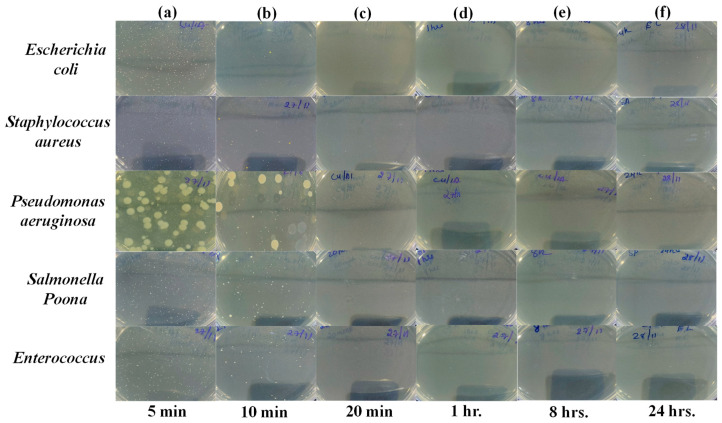
Images of Petri dishes used in agar diffusion method for “PLA-Cu-Al Sheet” against “*Escherichia coli*, *Staphylococcus aureus*, *Pseudomonas aeruginosa*, *Salmonella* Poona, and *Enterococci*” during different time intervals. (**a**) 5 min, (**b**) 10 min, (**c**) 20 min, (**d**) 1 h, (**e**) 8 h, and (**f**) 24 h.

Images of Petri dishes used in the agar diffusion method for the “PLA-Cu-Al Sheet” against “*Escherichia coli*, *Staphylococcus aureus*, *Pseudomonas aeruginosa*, *Salmonella* Poona, and *Enterococci*” micro-organisms during different time intervals (i.e., 5 min, 10 min, 20 min, 1 h, 8 h, and 24 h) are given in Figure 8a–f.

Arriagada et al. [46] developed thermally reduced PLA–Graphene oxide sheets by varying the amount of graphene oxide from 3% to 10%. The antibacterial efficiency of the prepared sheets was tested against *Escherichia coli* and *Staphylococcus aureus*. The authors reported 100% bacterial reduction with PLA and 5% pure graphene oxide, and PLA with 10% thermally reduced graphene oxide after 24 h. Lee et al. [47] prepared three types of coatings on stainless steel using zirconium oxide, zinc oxide, and titanium oxide and reported 81.2% and 72.4% killing efficiency against *Escherichia coli* and *Staphylococcus aureus*, respectively. Krumdieck et al. [48] used stainless steel doped with titanium oxide and achieved 99.9% efficiency against *Escherichia coli* after 4 h under UV region. Cao et al. [49] used a modified antibacterial peptide on a steel surface and tested its efficiency against two types of marine bacteria (*Vibrio natriegens* and *Citrobacter farmer*) and reported 99.79% and 99.33% efficiency against these bacteria, respectively, after 24 h.

Based on the previously reported data and the results of the present study, it is reasonable to conclude that 3D printed PLA-Cu-SS and PLA-Cu-Al composites can be effectively used for antimicrobial applications against *Escherichia coli*, *Staphylococcus aureus*, *Pseudomonas aeruginosa*, *Salmonella* Poona, and *Enterococci* [50].

## 3. Materials and Methods

**Figure 9:**
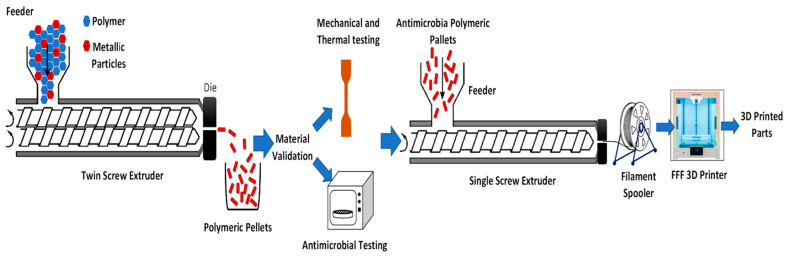
Process flow diagram of the experimental setup.

Leftover recycled PLA was used as a matrix for the reinforced composite [4,51]. The material used for the current study was in 3D printing filament form. Filaments are in the form of metal powders encased in a binder of environmentally friendly, biodegradable, and carbon-neutral polymers polylactide (PLA) [43]. Several fabrication techniques have been used for additive manufacturing; however, material extrusion (ME) is the most widely used technique, in which material filament is extruded and heated up to the polymer’s melting point [1]. Easy operating conditions, process safety, cost-effectiveness, and minimal waste production are the significant advantages of the ME process [52]. Each set of bimetallic composites was prepared by a known composition of metal particles and insulated by a polymeric material, i.e., PLA, to make the bimetallic compounds nonconductive. Additionally, 3D-printed strips of different materials (Cu-SS and Cu-Al) were printed side by side and over a polymeric substrate, i.e., PLA. Two or more non-conductive metallic materials could be used in different combinations, such as 3D printed strips that are produced side by side or can be made by any other technology, such as particles mixed with polymeric-based material. Moreover, one or more spray-based bimetallic compounds can be applied on conductive or insulated substrates. The developed bimetallic compound can be sprayed using two or more materials and conductive or insulated substrates. The process flow diagram for the experimental procedure is given below in Figure 9.

**Figure 10:**
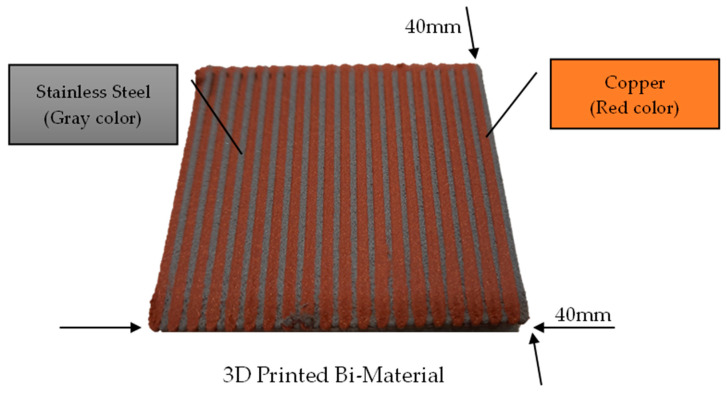
3D printed sample of copper and stainless steel.

Generally, the two sets of material combinations have been made using the fused filament fabrication 3D printing process to produce side-by-side dual materials straps. Set 1 is Cu-PLA and SS-PLA, which consist of 90 wt.% of copper, and 85 wt.% of SS 17-4, with a density of 4.7 g/cc and 3.0 g/cc, respectively. Set 2 is Cu-PLA and Al-PLA, which consists of 65 wt.% of Al, with a density of 1.54 g/cc. Figure 10 illustrates a side-by-side 3D printed sample of a dual material.

Pure stainless steel does not have any remarkable antimicrobial characteristics; however, its properties can be improved by introducing different metal particles into the steel matrix or altering its surface chemistry through various methods, e.g., electrodeposition, surface coatings, and heating treatments, as described in the previous studies [28,53,54].

**Figure 11:**
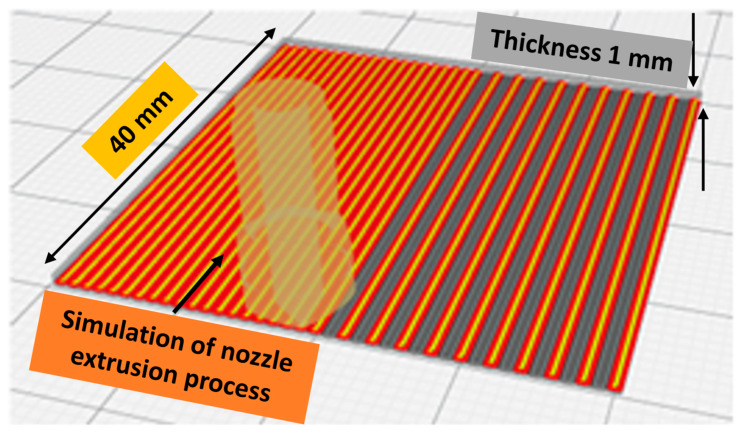
Slicing simulation of 3D printing process of dual-material with slicing characteristics determined using the Cura slicer.

Each sample set comprises metal powder and polymer matrix mixed by an extruder [55]. The spooler pulls the extruded material from the extrusion nozzle at a constant linear travel rate while optionally allowing spooling of the material, where the spool speed is usually more significant than the tension roller’s speed. The dimensions of the sample are 40 mm × 40 mm × 1 mm, and it was designed using CAD software Fusion 360 (Release V.2.0) and sliced using Ultimaker Cura 4.10 (Utrecht, The Netherlands), an open-source slicing application for 3D printers [56]. The 3D printer Ultimaker UM S5 was used to print the samples to be tested. It was specially designed to print using composite materials on the 3D printer Ultimaker S5 at a maximum temperature of 300 °C. The 3D printer was developed for the composite materials of third-party material suppliers that can wear out the standard Ultimaker UM S5 Core Head AA. Therefore, they should be printed using the print core CC, so we applied a hardened steel nozzle sized at 0.6 mm using Ultimaker print core CC 0.6. The printing nozzle temperature was maintained at 210 °C, while the printing bed temperature was kept at 50 °C; the bed was covered using a layer of blue painter’s tape or glue sticks to achieve maximum adhesion, and the printing flow rate was set at 135%. The filament was preheated at 60 °C using a warming chamber placed before the feeding gear to minimize any filament bending as it came off the spool. As the filament passed through the warmer, the memory of the filament was reset for ease of printing [57]. Figure 11 illustrates the simulation of the 3D printing process with slicing characteristics determined using the Cura slicer. Generally, the 3D printed sample was selected to fit within the testing containers, whereas thickness was considered to maintain the proper stiffness of samples to avoid any excessive deformation that could affect their condition during and/or after the printing process. The operating parameters of the Cura slicer were maintained as follows: 0.2 mm layer height, infill pattern lines, 100% infill density, no support, no adhesion type with a speed of 45 mm/s, and the fan cooling was kept at 100%. The back side of the sample was softer than its front side due to the smooth surface of the printing bed; therefore, the smooth side of the 3D printed specimen was always used for conducting antimicrobial testing.

### Antimicrobial Testing and Standards

The antimicrobial activity of materials can be tested using different methods such as agar-based and diffusion methods. These methods can be further classified as disc diffusion [58], well diffusion [59], disc volatilization [60], agar spot diffusion, and parallel streak method [61,62]. The agar disc diffusion method is one of the oldest and most common practicing methods for routine testing [63]. The Clinical and Laboratory Standards Institute has published several methods for evaluating bacteria and yeast [64]. These procedures are regarded as the antimicrobial susceptibility testing (AST) norms. They are used to determine the minimum inhibitory concentration (MIC) for various bacteria on agar plates (agar dilution), in broth microdilution, or macrodilution medium. The method has been modified to test a variety of pathogens, including *Streptococci*, *Haemophilus influenzae*, *Haemophilus parainfluenzae*, *Neisseria gonorrhoeae*, and *Neisseria meningitidis*, using particular culture media, various incubation conditions, and interpretive criteria for inhibition zones, even though this method cannot accurately test all the fastidious microbes [64,65]. The antimicrobial activity of surfaces can be analyzed by following the three major standards suggested by Japan (JIS Z2801:2010) [66], Europe (ISO 22196:2011) [33], and recently the United States (US EPA) [67]. According to the Japanese standard, antimicrobial activity is an inhibition of the growth of bacteria on the surface of the material. However, ISO 20743 [68] considers both inhibition and death of bacteria on the surfaces [69]. These methods are adapted to assess the antimicrobial activity and efficiency of plastic, nonporous, and hard surfaces. The standard proposed by the United States Environmental Protection Agency (EPA) has gained more attention than the abovementioned methods because it provides the equations and conditions to test antimicrobial activity and normalized procedures to study the effect of biocidal cleaning liquids on nonporous surfaces. A material is said to be a sanitizer if 99.9% of bacteria are killed within 1 h. On the other hand, the Japanese and European standards do not set an antibacterial activity threshold but rather give a framework for standardized antimicrobial activity quantification; the range of items they cover is more significant, making benchmarking more challenging [24,70]. Another standard, ISO 22196 [33], has been developed to test the activity of bacteria and viruses on plastic surfaces for a time interval of 24 h. Further modifications have been made to this method to make it applicable to other nonporous surfaces. It is an excellent approach to establishing the antimicrobial activity of a surface. This has become one of the industry standards among several tests for the antibacterial activity of surfaces [59]. The quantitative and precise assessment of antimicrobial surfaces (e.g., plastics, metals, and ceramics) is conducted according to the JIS Z 2801 standard [66]. This method has various real-world applications in different fields, ranging from healthcare centers to household consumer companies. This is the most adopted method in the United States, and it has become an industry standard; however, it complicates identifying an ideal control surface [71]. The BS ISO 22196:2011 standard [33] measures the antimicrobial activity on plastic surfaces and paint films that are not light-activated. This standard commonly uses Escherichia coli or Staphylococcus aureus [72]. The bacteria were measured using the pour plate technique according to CCFRA 1.1.4:2003 [34]. The entire test method was developed in-house. The antimicrobial activity of the prepared samples was tested against common bacteria that were obtained from certified reference materials, which were preserved in the lab as QC controls. The testing protocol was devised according to ISO 22196:2011 [33], and microbiology analytical methods were derived from CCFRA:1:1:4:2003 [35,36].

The investigation results have been analyzed mainly based on the data collected from the samples regarding the antimicrobial resistance of the developed composites, reflecting the significant improvements of the antimicrobial resistance compared to the individual materials used to formulate the investigated composites. A detailed statistical analysis of the current study results has been tested and analyzed using the statistical *t*-test and the ANOVA method using Minitab software (Release 21.3.1), which is demonstrated in Appendix A.

## 4. Conclusions

The antimicrobial properties of hybrid composites of polylactide acid (PLA) have been improved by adding metallic particles of copper (90 wt.%), stainless steel (85 wt.%), aluminum (65 wt.%), and a known amount of PLA via 3D printing. Hybrid composites of these metallic particles were prepared using a printing technique, which is an efficient, cost-effective, and rapid method to introduce metal particles in the polymer matrix, which can be enhanced by using natural material, and where the recycled plastic waste can be used as a matrix to develop the antimicrobial composite. The prepared hybrid composites were tested against Gram-positive and Gram-negative bacteria at different time intervals. They compared the results with a plastic sheet taken as a reference. Both sets of composite materials exhibited excellent antibacterial activity against tested bacteria. They achieved more than 99% bacterial reduction just after 10 min, and no significant difference between the performance of these composites was observed. Results presented here are considerably superior to those previously published due to the higher amount of metallic particles and the preparation technique.

Furthermore, the development of hybrid metallic composites for antibacterial applications using 3D printing techniques considerably enhanced their characteristics and efficiency; therefore, this technology may be helpful for further research and biomedical applications. Moreover, this technology can be used in food packaging, textile, space, and other applications, e.g., recycling waste materials and incorporating nanotechnology for improved performance. Additionally, the development of hybrid metallic composites for antibacterial applications using the 3D printing technique considerably enhanced their characteristics and efficiency; therefore, this technology may be helpful in further research and biomedical applications. Moreover, this technology can be used in food packaging, textile, space, and other applications, e.g., recycling waste materials and incorporating nanotechnology for improved performance.

## Figures and Tables

**Table 1:** Bacterial count of all bacteria on “Control Sheet” during different time intervals.

Sample	Challenged Bacteria	Inoculum	Bacterial Count					
			5 min	10 min	20 min	1 h	8 h	24 h
Control Sheet	*Escherichia coli*	9700	9100	8500	7700	6900	6100	1850
	*Staphylococcus aureus*	6300	5700	5300	4850	4200	2700	52
	*Pseudomonas aeruginosa*	8200	7700	7200	6800	5100	3300	51
	*Salmonella* Poona	9500	8800	8150	7900	7100	3100	1100
	*Enterococci*	7400	6100	5800	5200	4400	3100	320

**Table 2:** The bacterial count of all bacteria on “PLA-Cu-SS Sheet” during different time intervals.

Sample	Challenged Bacteria	Inoculum	Bacterial Count					
			5 min	10 min	20 min	1 h	8 h	24 h
PLA-Cu-SS	*Escherichia coli*	9700	115	43	1	1	1	1
	*Staphylococcus aureus*	6300	132	33	1	1	1	1
	*Pseudomonas aeruginosa*	8200	106	30	1	1	1	1
	*Salmonella* Poona	9500	124	45	1	1	1	1
	*Enterococci*	7400	141	29	1	1	1	1

**Table 3:** Bacterial count of all bacteria on “PLA-Cu-Al Sheet” during different time intervals.

Sample	Challenged Bacteria	Inoculum	Bacterial Count					
			5 min	10 min	20 min	1 h	8 h	24 h
PLA-Cu-Al	*Escherichia coli*	9700	152	61	1	1	1	1
	*Staphylococcus aureus*	6300	165	44	1	1	1	1
	*Pseudomonas aeruginosa*	8200	134	46	1	1	1	1
	*Salmonella* Poona	9500	163	59	1	1	1	1
	*Enterococci*	7400	173	38	1	1	1	1

## Data Availability

Available upon request.

## Appendix A. Results Statistical Analysis

A *t*-test using Minitab software was conducted in the analysis for the 5 min and 10 min results, significantly impacting the study results for the Cu-SS and the Cu-Al composites. A *t*-test aims to determine whether the difference between the means of two groups is statistically significant. In other words, it is used to test whether the observed difference in means is due to chance or whether it is a natural, meaningful difference. In general, the *t*-test is a statistical test used to compare the means of two groups and determine whether the difference between them is statistically significant. It is a parametric test, assuming the analyzed data are typically distributed [73]. The *t*-test works by comparing the difference between the means of two groups to the variability within the groups. Specifically, it calculates the t-statistic, which is the difference between the means of the two groups divided by the standard error of the difference.

For the 5 min group of results:

*t*-Test is considered for two independent means [74] significance level: 0.05 and for the one-tailed test.

*Cu-SS Composite*			
*N*_1_: 5			
*df*_1_ = *N* − 1 = 5 − 1 = 4			
*M*_1_: 98.44			
*SS*_1_: 0.69			
*s*^2^_1_ = *SS*_1_/(*N* − 1) = 0.69/(5 − 1) = 0.17			
*Cu-Al Composite*			
*N*_2_: 5			
*df*_2_ = *N* − 1 = 5 − 1 = 4			
*M*_2_: 98.02			
*SS*_2_: 0.9			
*s*^2^_2_ = *SS*_2_/(*N* − 1) = 0.9/(5 − 1) = 0.22			
***t*-value Calculation**			
*s*^2^*_p_* = ((*df*_1_/(*df*_1_ + *df*_2_)) * *s*^2^_1_) + ((*df*_2_/(*df*_2_ + *df*_2_)) * *s*^2^_2_) = ((4/8) * 0.17) + ((4/8) * 0.22) = 0.2			
*s*^2^_*M*1_ = *s*^2^*_p_*/*N*_1_ = 0.2/5 = 0.04			
*s*^2^_*M*2_ = *s*^2^*_p_*/*N*_2_ = 0.2/5 = 0.04			
*t* = (*M*_1_ − *M*_2_)/√ (*s*^2^_*M*1_ + *s*^2^_*M*2_) = 0.42/√0.08 = 1.48			
Where *N* = no. of samples; *M* = mean value; *s* = standard deviation; *df* = degrees of freedom in the source; *ss* = sum of squares due to the source; Source = source of the variation in the data; *Ms* = mean sum of squares due to the source; *F* = the *F*-statistic; *p* = the *p*-value.

The statistical results revealed that the *t*-value is 1.47832. The *p*-value is 0.088789, so the conclusion is that the results are not significant at *p* < 0.05. Accordingly, the *t*-test analysis has been executed again but at a significance level: 0.1, to figure out whether the possibility is to be accepted or not. The investigation by Mintab demonstrated that the statistical analysis depicted that the *t*-value is 1.47832. The *p*-value is 0.088789, so the result is significant at *p* < 0.10.

On the other hand, regarding the statistical analysis for the 10 min results using the same conditions above, the *t*-test results showed that:

*Cu-SS Composite*			
*N*_1_: 5			
*df*_1_ = *N* − 1 = 5 − 1 = 4			
*M*_1_: 99.56			
*SS*_1_: 0.01			
*s*^2^_1_ = *SS*_1_/(*N* − 1) = 0.01/(5 − 1) = 0			
*Cu-Al Composite*			
*N*_2_: 5			
*df*_2_ = *N* − 1 = 5 − 1 = 4			
*M*_2_: 99.4			
*SS*_2_: 0.02			
*s*^2^_2_ = *SS*_2_/(*N* − 1) = 0.02/(5 − 1) = 0.01			

***t*-value Calculation**		
*s*^2^*_p_* = ((*df*_1_/(*df*_1_ + *df*_2_)) * *s*^2^_1_) + ((*df*_2_/(*df*_2_ + *df*_2_)) * *s*^2^_2_) = ((4/8) * 0) + ((4/8) * 0.01) = 0		
*s*^2^*_M_*_1_ = *s*^2^*_p_*/*N*_1_ = 0/5 = 0		
*s*^2^*_M_*_2_ = *s*^2^*_p_*/*N*_2_ = 0/5 = 0		
*t* = (*M*_1_ − *M*_2_)/√ (*s*^2^*_M_*_1_ + *s*^2^*_M_*_2_) = 0.17/√0 = 3.93		

The *t*-value at 10 min is 3.93458, and the *p*-value is 0.002164, so the result is significant at *p* < 0.05, contrary to the 5 min findings.

ANOVA (Analysis of Variance) has been used to interpret the results statistically; it is a statistical test used to analyze whether there is a significant difference between the means of two or more groups. It is a parametric test that assumes that the data is typically distributed and the variances are equal across the groups [75]. The goal of ANOVA is to determine if the variance between groups is larger than the variance within groups. It is important to note that ANOVA assumes that the data is usually distributed and that the variances are equal across groups. Due to significant antimicrobial enhancement encountered, Minitab has been used to conduct ANOVA analysis for the two groups’ results at 5 min and 10 min for the developed composites, Cu-SS, and the Cu-Al, at significance Level 0.05, as follows:

	**Cu-SS Composite**	**Cu-Al Composite**
Mean	98.44	98.024
Standard deviation	0.4142	0.4736

**Result Details**				
**Source**	***SS***	***df***	***MS***	*F* = 2.18544
Between treatments	0.4326	1	0.4326		
Within treatments	1.5837	8	0.198		
Total	2.0164	9			

It has been found that the f-ratio value is 2.18544 and the *p*-value is 0.177579, so the result is not significant at *p* < 0.05. The analysis has been repeated at a significance level: 0.1, and the results demonstrated that the f-ratio value is 2.18544 and the *p*-value is 0.177579, so again, the results are not significant even at *p* < 0.10. In addition, the 10 min results have been analyzed using the ANOVA test by Minitab software at a significance level: 0.05, which depicted the following:

	**Cu-SS Composite**	**Cu-Al Composite**
Mean	99.562	99.396
Standard deviation	0.0606	0.0723

**Result Details**				
**Source**	***SS***	***df***	***MS***	*F* = 15.4809
Between treatments	0.0689	1	0.0689		
Within treatments	0.0356	8	0.0044		
Total	0.1045	9			

It has been estimated that the f-ratio value is 15.4809 and the *p*-value is 0.004329, so in conclusion, the result is significant at *p* < 0.05, which is opposite to the 5 min results.
